# Enzyme kinetic approach for mechanistic insight and predictions of *in vivo* starch digestibility and the glycaemic index of foods

**DOI:** 10.1016/j.tifs.2021.11.015

**Published:** 2022-02

**Authors:** Peter J. Butterworth, Balázs H. Bajka, Cathrina H. Edwards, Frederick J. Warren, Peter R. Ellis

**Affiliations:** a Biopolymers Group, Departments of Biochemistry and Nutritional Sciences, Faculty of Life Sciences and Medicine, King's College London, Franklin-Wilkins Building, 150 Stamford Street, London, SE1 9NH, UK; b Quadram Institute Bioscience, Rosalind Franklin Road, Norwich Research Park, Norwich, NR4 7UQ, UK

**Keywords:** Alpha-amylase, Starch digestion, Enzyme kinetics, Resistant starch, Gene copy number, Metabolic significance, AMY1, human salivary α-amylase gene, AMY2, human pancreatic α-amylase gene, BMI, body mass index, CE, catalytic efficiency, CVD, cardiovascular disease, Fto, alpha-oxoglutarate-dependent dioxygenase gene, GI, glycaemic index, GIT, gastrointestinal tract, GL, glycaemic load, GLUT2, glucose transporter 2, HI, hydrolysis index, IC_50_, inhibitor concentration causing 50% inhibition, LOS, logarithm of slope plot, RDS, rapidly digestible starch, RS, resistant starch, SCFAs, short chain fatty acids, SDS, slowly digestible starch, SGLT1, sodium-dependent glucose co-transporter, XRD, X-ray diffraction

## Abstract

**Background:**

Starch is a principal dietary source of digestible carbohydrate and energy. Glycaemic and insulinaemic responses to foods containing starch vary considerably and glucose responses to starchy foods are often described by the glycaemic index (GI) and/or glycaemic load (GL). Low GI/GL foods are beneficial in the management of cardiometabolic disorders (e.g., type 2 diabetes, cardiovascular disease). Differences in rates and extents of digestion of starch-containing foods will affect postprandial glycaemia.

**Scope and approach:**

Amylolysis kinetics are influenced by structural properties of the food matrix and of starch itself. Native (raw) semi-crystalline starch is digested slowly but hydrothermal processing (cooking) gelatinises the starch and greatly increases its digestibility. In plants, starch granules are contained within cells and intact cell walls can limit accessibility of water and digestive enzymes hindering gelatinisation and digestibility. *In vitro* studies of starch digestion by α-amylase model early stages in digestion and can suggest likely rates of digestion *in vivo* and expected glycaemic responses. Reports that metabolic responses to dietary starch are influenced by α-amylase gene copy number, heightens interest in amylolysis.

**Key findings and conclusions:**

This review shows how enzyme kinetic strategies can provide explanations for differences in digestion rate of different starchy foods. Michaelis-Menten and Log of Slope analyses provide kinetic parameters (e.g., *K*_*m*_ and *k*_*cat*_*/K*_*m*_) for evaluating catalytic efficiency and ease of digestibility of starch by α-amylase. Suitable kinetic methods maximise the information that can be obtained from *in vitro* work for predictions of starch digestion and glycaemic responses *in vivo*.

## Highlights

• Michaelis-Menten kinetics determines catalytic efficiency of amylase action on starch.
• Kinetic parameters *K*_*m*_ and *k*_*cat*_*/K*_*m*_ are useful for estimating ease of digestibility.
• Log of slope analysis can quantify starch fractions digested at different rates.
• Categorisation of Resistant Starch can be based on types of interaction with amylase.
• Enzyme kinetic studies of amylolysis are useful for predicting postprandial glycaemia.

## Graphical abstract

**Figure:**
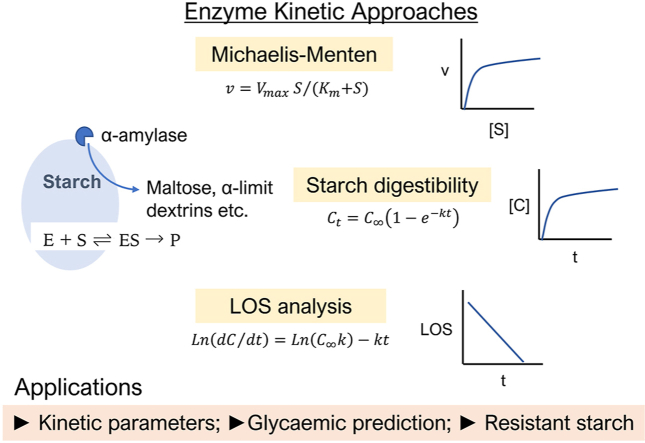

## 1 Introduction

Starch is a principal source of dietary energy for humans accounting for 35–70% of the total energy intake in the form of glucose in modern Western diets (Copeland, 2016). It is consumed in foods derived from cereal crops such as wheat and rice, and from plant underground storage organs such as potatoes and yams together with many legume seeds (e.g., peanuts, peas, chickpeas and common bean varieties). Starch is packaged in granules found within the plant cell bounded by cell walls along with variable amounts of lipid and protein, which limit the general accessibility of water and enzymes thus affecting starch digestion in the gastrointestinal tract (GIT) (Edwards, Ryden, Mandalari, Butterworth, & Ellis, 2021; Edwards, Grundy, Grassby, Vasilopoulou, Frost et al., 2015; Edwards, Warren, Campbell, Gaisford, Royall et al., 2015; Petropoulou et al., 2020). Plant cell walls are polysaccharide bioassemblies of non-digestible carbohydrates plus smaller and variable amounts of other compounds such as phenolics and protein. This fraction of the plant material, known as ‘dietary fibre’, is not digested by host enzymes in the proximal human gut but potentially fermented by colonic microbiota (Grundy et al., 2016).

**Fig. 1:**
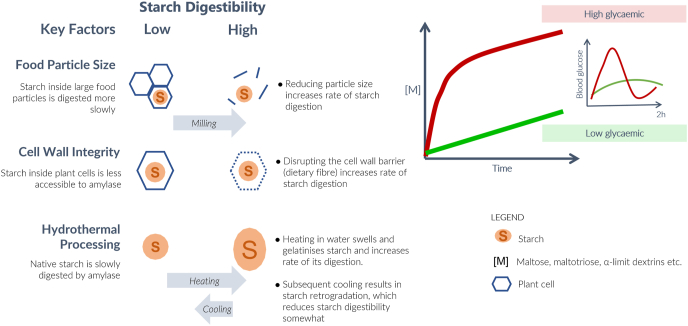
Some key factors that affect starch digestion kinetics and postprandial glycaemia. Main factors include food structure, and hydrothermal (cooking in water) and mechanical (milling) processing. Low and high rates of starch digestion are linked to low and high glycaemic responses, respectively. Processing increases the susceptibility of starch in plant foods to α-amylase (i.e., increases the rate and extent of digestion). Gelatinised starch (post-cooking) is more susceptible to amylolysis, but starch that remains in the native state or has become retrograded (following cooling and storage) is significantly less susceptible to amylase action. For further details of factors that affect starch digestion see Lovegrove et al. (2017).

Typically, native (raw) starch represents only a small percentage of the total starch intake. Some raw starch granules from bananas, peanuts and uncooked vegetables may be eaten, but most dietary starch originates from bread, pasta, rice, potato and yams that have been hydrothermally processed (by cooking in water). This treatment significantly increases starch gelatinisation and its digestibility (Dhital, Warren, Butterworth, Ellis, & Gidley, 2017; Roder et al., 2009). Starch in biscuits (cookies in the USA) can be resistant to amylolysis because biscuit/cookie recipes contain low water levels (Roder et al., 2009). Mechanical food processing, such as milling, disrupts plant tissues and cell walls. This increases susceptibility of starch to gelatinisation during hydrothermal processing and facilitates the access of starch to digestive enzymes by removing the cell wall barrier (Dhital et al., 2017; Edwards, Grundy et al., 2015; Edwards, Warren et al., 2015) (Fig. 1).

An account of starch digestion in the upper gut appears in a recent review (Brownlee, Gill, Wilcox, Pearson, & Chater, 2018). The first stage in amylolysis is hydrolysis of the polyglucan chains in starch catalysed by salivary and pancreatic α-amylases with production of maltose, maltotriose and α-limit dextrins predominantly (Roberts & Whelan, 1960). However, the compact granular structure and crystallinity of native (uncooked) starch granules means that amylolysis proceeds slowly and to a limited degree (Slaughter, Ellis, & Butterworth, 2001). Complete digestion of amylolytic products by epithelial membrane enzymes, sucrase-isomaltase and maltase-glucoamylase (Nichols et al., 2003), yields plentiful amounts of glucose, which is absorbed into the hepatic portal vein via the SGLT1 and GLUT2 transporters (Kellet & Brot-Laroche, 2005). Glucose can then be metabolised by all tissues and organs but is an essential metabolic fuel for the central nervous system and for glycolytic tissues (e.g., kidney medulla) and cells such as erythrocytes. The ability to control fire and the development of cooking by our hominin ancestors would have led to higher yields of glucose from ingested starch. Arguably this became very important as growth in brain size in humans evolved and lifestyles changed from hunter-gatherer to settled agrarian ones (Butterworth, Ellis, & Wollstonecroft, 2016; Hardy & Kubiak-Martens, 2016).

Enzyme kinetic studies, if well performed, are excellent indicators of the rate and extent of starch digestibility (amylolysis). Such findings are valuable for predicting potential behaviour *in vivo* (see Sections 5, 6). The different *in vitro* methods used for simulating food digestion and absorption in the GIT is also an important topic, but this is covered elsewhere (Brodkorb et al., 2019; Lefebvre et al., 2015; Minekus et al., 2014; Wickham, Faulks, Mann, & Mandalari, 2012). Values for postprandial glycaemia, determined from *in vivo* nutritional studies, are often considered to be the most reliable guides of dietary responses to starch and other digestible carbohydrates. It is well understood however that the results obtained for glycaemia can be subject to other variables such as the metabolic state of individuals and previous food intake. We hope that our article will help workers in coming to a better understanding of enzyme kinetics as applied to starch digestion and its usefulness and limitations.

## 2 Human α-amylases

Interest in salivary α-amylase has increased greatly. A nineteenth century demonstration that human saliva contains an agent that catalyses starch breakdown was important for studies of enzyme catalysis (Butterworth, Ellis, & Warren, 2011). Kinetic and molecular properties of salivary amylase have remained of interest and its 3D structure has been determined and the nature of the catalytic site established (Ramasubbu, Paloth, Luo, Brayer, & Levine, 1996). Similar detailed structural information is also available for human and porcine pancreatic amylases (Brayer et al., 2000; Zhang et al., 2016).

### 2.1 Gene copy numbers of salivary and pancreatic amylase and possible metabolic significance

The contribution of salivary amylase to the digestion of starch is of considerable conjecture (Butterworth, Warren, & Ellis, 2011). For instance, it has been assumed that the quantity of starch digested during the relatively short period that a food bolus remains in the mouth will be negligible compared with the amount that is hydrolysed by pancreatic amylase. Also, gastric acidity would inactivate amylase. There is evidence, however, that catalytically active salivary amylase can be detected in the duodenum (Fried, Abramson, & Meyer, 1987; see section *2.2.*).

Humans possess separate but related genes denoted as *AMY1* and *AMY2* that code for salivary and pancreatic α-amylases, respectively. The number of copies of the genes in individuals is variable (Groot et al., 1989; Iafrate et al., 2004; Perry et al., 2007). Considerable interest in salivary amylase arose following reports that *AMY1* copy number, which can range from 2 to 20 or so, tends to be higher in populations that consume large amounts of starch (Iafrate et al., 2004; Perry et al., 2007). Numerous accounts have appeared of studies of links between gene copy number, metabolic responses to dietary starch and risk of developing obesity (Mandel & Breslin, 2012; Nakajima, Nemoto, Kakei, Fuchigami, & Munakata, 2011) but the reports are contradictory. Some suggest a low copy number of *AMY1* is associated with raised BMI and/or increased risk of insulin resistance (Choi et al., 2015; Falchi et al., 2014; Mandel & Breslin, 2012). Other studies however have failed to establish clear relations of *AMY1* copy number and BMI (Alberti et al., 2015; Atkinson, Hancock, Petocz, & Brand-Miller, 2018; Carpenter et al., 2015; Poole et al., 2019; Rukh, Ericson, Andersson-Assarsson, Orho-Melander, & Sonestedt, 2017; Usher et al., 2015; Valsesia et al., 2019), although low salivary amylase was associated with a preference, in men, for diets high in sugar content (Tarragon, Stein, & Meyer, 2018). Some of the contradictions could arise from the methodology used by different investigators that may have been unable to distinguish between *AMY1* and *AMY2* (Carpenter et al., 2015). The pancreas contains two distinct but related amylase genes *AMY2A* and *AMY2B,* the copy numbers of which differ in different populations with two copies of each gene being a frequent pattern. It seems that the copy numbers of *AMY2* genes may associate with those of *AMY1* (Carpenter et al., 2015).

It appears that links between copy number and obesity depend on dietary starch intake (Atkinson et al., 2018). Amongst individuals consuming considerable amounts of starch, those with high *AMY1* copy numbers produced a modestly higher postprandial glycaemia than those with low copy numbers (Atkinson et al., 2018; Carpenter et al., 2015). East Asian populations tend to have higher copy numbers than those of European Caucasians and that higher catalytic activity in saliva accompanies the higher gene copy number (Atkinson et al., 2018; Carpenter et al., 2015) i.e., multiple copies of the gene are expressed. Glycaemic responses to starchy foods were greatest in a group of individuals with high *AMY1* copy number compared with a low copy number group (Atkinson et al., 2018). However, the latter group was associated with higher breath methane levels, suggesting differences in microbial metabolism in the large intestine between the groups. Related to this finding, it has been shown that *AMY1* copy number affects the oral and gut microbiome compositions (Poole et al., 2019). The physiological significance of gene copy number is clearly an important factor for starch digestion kinetics, but opinions on the subject continue to differ. Further study is required for resolution of the controversies.

### 2.2 Digestion of dietary starches

Chewing of food and mixing with saliva in the mouth initiates starch breakdown by salivary amylase, a process that continues during bolus formation and swallowing. The contribution made by salivary amylase to starch digestion can be underestimated because of an assumption that once a bolus is swallowed, the low pH of the gastric contents will inhibit amylase activity, the optimum pH of which lies between 6.5 and 7 (Walker & Whelan, 1960). However, salivary amylase bound to starch and/or oligosaccharides is protected from acid-induced inactivation and can be detected in intestinal fluid (Fried et al., 1987; Rosenblum, Irwin, & Alpers, 1988). Up to 80% of the starch in white bread formed in a bolus from mixing of the food with saliva, can be digested within 30 min of residence in the stomach (Freitas, Le Feunteun, Panouille, & Souchon, 2018). The authors of this article cited a 1926 publication of a human study that reported extensive digestion in the stomach of starch from mashed potatoes and from wheat bread (Bergheim, 1926). On reaching the small intestine any remaining starch is then digested by pancreatic α-amylase. However, if starch remains encapsulated within a food matrix, such as plant tissue, it can be shielded from amylase action (Fig. 1) thereby escaping digestion (Dhital et al., 2017; Edwards, Grundy et al., 2015; Edwards, Ryden et al., 2021; Edwards, Warren, Milligan, Butterworth, & Ellis, 2014; Grundy et al., 2016; Petropoulou et al., 2020).

The digestion of starch-rich foods is followed by postprandial rises in blood glucose concentration and secretion of insulin. For certain starch-rich foods, postprandial rises in blood glucose and insulin occur rapidly causing a peak in concentration. Foods with identical starch contents can produce glycaemic and insulinaemic responses that differ widely (Augustin et al., 2015; Dhital et al., 2017; Edwards, Grundy, et al., 2015; Jenkins et al., 1981). Therefore, determination of the glycaemic index (GI) and/or glycaemic load (GL), which takes account of the quantity of digestible carbohydrate in the ingested food, have been used to classify different foods (Augustin et al., 2015; Jenkins et al., 1981, 2002).

Several reports have suggested that diets classified as low GI are associated with a reduced risk of developing obesity with its propensity for cardiovascular disease (CVD), insulin resistance and type-2 diabetes (Augustin et al., 2015; Brouns et al., 2005; Jenkins et al., 2002; Livesey et al., 2019; Livesey, Taylor, Hulshof, & Howlett, 2008). Obesity is also linked with the development of many types of cancer (Hooper et al., 2017). In humans, expression of the fat mass and obesity-associated gene (*Fto*) is known to affect appetite and the mass of body fat. Studies in mice have shown that high GI diets result in increased *Fto* expression that can lead to raised levels of body fat (Sideratou et al., 2018). Diets containing low GI/or GL foods tend to avoid rapid and exaggerated excursions in glycaemia and insulinaemia and are less likely therefore, to raise the risks of developing CVD and type 2 diabetes in the long term (Augustin et al., 2015; Bhupathiraju et al., 2014; Jenkins et al., 2002; Livesey et al., 2019; Livesey et al., 2008; Wolever et al., 1992). Moreover, low GI/GL diets are known to be advantageous in diabetes management by improving glycaemic control and blood lipid profiles in people with type 2 diabetes (Augustin et al., 2015; Jenkins et al., 2002; Wolever et al., 1992).

Undigested starch, termed ‘resistant starch’ (RS), reaches the colon together with other non-digestible carbohydrates, notably non-starch polysaccharides of plant cell walls (fibre) (Gibson & Roberfroid, 1995; Topping & Clifton, 2001). This material is metabolised by the microbiome to short-chain fatty acids (SCFAs), primarily acetate, propionate and butyrate (Gibson & Roberfroid, 1995; Topping & Clifton, 2001). Of the SCFAs, butyrate is an important fuel for colonocytes, acetate is used in hepatic lipogenesis and as an energy substrate in muscle, and propionate can be a source of phosphoenolpyruvate that may enter gluconeogenesis (Canini et al., 2011; Chambers, Preston, Frost, & Morrison, 2018; Gibson & Roberfroid, 1995; Topping & Clifton, 2001). All SCFAs seem to be important for the maintenance of the mucosal cells and for stimulating the release of gut hormones that act on the pancreas, improving insulin sensitivity, and on the hypothalamus to affect appetite and blood pressure regulation. Also, SCFAs provide protection against colorectal cancer. Propionate appears in the blood circulation and is likely to be particularly important for initiating hormonal responses. (Canini et al., 2011; Chambers et al., 2018; Gibson & Roberfroid, 1995; Topping & Clifton, 2001).

Hence there are good health reasons for seeking ways to predict the postprandial responses to starch-rich foods, but measurements on human subjects are expensive to perform and results can be complicated by extraneous factors causing literature reports of GI for similar foods to differ somewhat (Brouns et al., 2005). A recommended methodology for GI determination is available, which details good practice and treatment of experimental data to minimise anomalies in the results from different laboratories (Brouns et al., 2005). Nevertheless, alternative *in vitro* strategies for assessing starch digestibility are valuable, not just for evaluating how starch-containing foods affect postprandial metabolism, but also for providing insight into molecular mechanisms of amylolysis (Baldwin et al., 2015; Butterworth et al., 2011; Dhital et al., 2017; Edwards et al., 2014; Edwards, Ryden et al., 2021). Perusal of the literature reveals that methods for measuring the digestion of starch by amylase are quite numerous, but many are based on determination of reducing sugars released by digestion and they can differ in sensitivity. Various methods for assaying amylase activity *in vitro* are described and compared in Appendix A (Online Supplementary Material).

## 3 Starch structure, properties and hydrolysis

Starch granules vary in size from approximately 0.1 to 100 μm in diameter depending on the botanical species (Pérez & Bertoft, 2010) and differ in shape between species and even within different tissues and cells of a single species (Copeland, 2016). Granules contain the polysaccharides amylose and amylopectin. Amylose accounts for ∼15–30% of total starch and is formed of an essentially linear chain of glucose residues in α-(1 → 4) glucosidic linkages with limited branching, but amylopectin (normally about 70–85% of the total) is a much larger molecule with an α-(1 → 4) linked backbone of glucose units and numerous α-(1 → 6) branch points (Pérez & Bertoft, 2010). Both polymers are composed entirely of glucose although some sugar residues, e.g., in potato starch, may be phosphorylated (Pérez & Bertoft, 2010) and affect the physiochemical properties of starch. Starch granules can also contain minor amounts of protein and lipid, the quantities of which vary between different botanical sources. Cereal starches contain free fatty acids and phospholipids that are bound to the amylose fraction, which can affect the rate at which starch is digested by α-amylase (Holm et al., 1983).

**Fig. 2:**
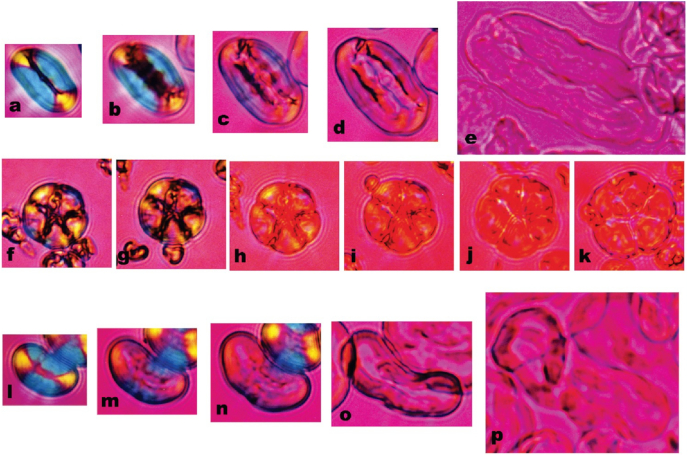
Micrographs of starch granules suspended in water subjected to heat treatment showing the loss of birefringence and gelatinisation. Wild type pea starch viewed at 30 (a), 60 (b), 62, (c). 64 (d) and 80 °C. Views of *r* mutant pea starch granules at 30 (f), 46 (g), 56 (h), 60 (i), 71 (j) and 80 °C (k). Views of *lam* mutant pea starch at 30 (l), 57 (m), 58 (n), 61 (o) and 80 °C (p). The granules were heated on the stage of a light microscope at a magnification of 400x under crossed polarisers in conjunction with a *lamba* plate. The different size images of the wild type and *lam* mutant pea starch reflect the marked changes in swelling during heating of the starch granules. Reproduced from Tahir et al. (2011), with permission.

Native starch granules are semi-crystalline with alternating bands of amorphous and crystalline material (Gérard, Planchot, Colonna, & Bertoft, 2000; Pérez & Bertoft, 2010). The crystalline regions are formed from densely packed amylopectin chains. The regular repeating double-helical structures of the crystalline material diffracts X-rays. Two general types of packing exist, giving rise to so-called A-type and B-type starches when examined by X-ray diffraction (Gérard et al., 2000; Pérez & Bertoft, 2010). Cereal starches are mostly A-type while tubers are B-type. Legume starches contain a mixture of A and B and are designated as C-type. The semi-crystalline native structure of raw starch is birefringent and exhibits a Maltese cross pattern when granules are viewed by cross-polarised light microscopy (Copeland, 2016; Pérez & Bertoft, 2010). B-type raw starches tend to be more resistant than A-type to digestion by amylase (Dhital et al., 2017; Roder et al., 2009). Birefringence disappears when the crystalline structure is disrupted during gelatinisation. Hydrothermal processing of starch and starch-containing foods results in gelatinisation, involving the uptake of water, swelling of the granules and leaching of mainly the amylose fraction, producing starch that is more amorphous and thus more digestible than native, raw starch (Dhital et al., 2017; Htoon et al., 2009; Lovegrove et al., 2017; Roder et al., 2009; Slaughter et al., 2001; Tahir, Ellis, Bogracheva, Meares-Taylor, & Butterworth, 2011) (Fig. 1, Fig. 2). Mechanical treatments, e.g., milling for wheat flour production, facilitates starch gelatinisation during the cooking because granules are more exposed to water in ruptured cells (Edwards et al., 2014; Edwards, Warren et al., 2015). This increase in starch bioaccessibility and gelatinisation increases susceptibility to amylolysis and therefore postprandial glycaemia (Dhital et al., 2017; Edwards et al., 2014; Lovegrove et al., 2017; Patel, Day, Butterworth, & Ellis, 2014; Roder et al., 2009; Tahir et al., 2011) (Fig. 1). Tahir and colleagues showed that increases in amylolysis in pea starches occurred in parallel with increases in amorphous material caused by increased starch swelling and gelatinisation during hydrothermal treatment (Tahir et al., 2011). The marked changes in granular structure during transformation from native to gelatinised pea starches can be seen in Fig. 2.

Disordered α-glucan chains in gelatinised starch may re-crystallise during cooling and storage particularly if exposed to chilled temperatures (Edwards, Veerbahu, Mason, Butterworth, & Ellis, 2021). This property of retrogradation occurs relatively rapidly in amylose but more slowly in amylopectin and the retrograded starch becomes resistant to digestion by α-amylase (Dhital et al., 2017; Englyst, Kingman, & Cummings, 1992; Htoon et al., 2009; Lovegrove et al., 2017; Patel et al., 2014; Patel et al., 2017). The resistant starch has a biological impact on the microbiome of the colon, particularly by favouring populations of microorganisms that are beneficial for colonic health. Thus, retrograded starch is an example of a dietary component with implications for improved health notably for the treatment/protection of inflammatory bowel disease, colorectal and other cancers and diabetes mellitus (Higgins & Brown, 2013; Lovegrove et al., 2017; Warren et al., 2018).

### 3.1 Resistant starch

Resistant starch (RS) is a physiological description of the fraction of dietary starch that enters the large intestine, i.e., material that escapes digestion during transit from the mouth to the terminal ileum (Dhital et al., 2017; Englyst et al., 1992; Lovegrove et al., 2017). Resistance to amylolysis can be a consequence of *inter alia*, the semi-crystallinity of raw starch, retrogradation, encapsulation within whole plant cells (due to structurally intact cell walls), complexation with protein and lipid or through chemical modification of starch molecules. The current classification of resistant starch as RS 1,2,3,4 etc, developed by Englyst takes account of these different types of RS and is a useful way of identifying many different forms of RS in the context of complex food matrices, but offers no mechanistic description for resistance (Englyst et al., 1992). The kinetic nature of the intransigence to digestion can be based on (a) access and binding of enzyme to starch, and (b) catalytic conversion of starch once amylase has become bound (Dhital et al., 2017). Thus, there is merit in classifying RS into just two categories *viz*: (a) causing interference of starch-enzyme binding or (b) inhibition of the catalytic event itself (Dhital et al., 2017). Interest in RS from a dietary viewpoint is that it not only slows *in vivo* digestion of starch with concomitant attenuation of spikes in glycaemia and insulinaemia, but also affects colonic microbiome composition with important benefits for colonic health (Canini et al., 2011; Chambers et al., 2018; Gibson & Roberfroid, 1995; Topping & Clifton, 2001).

## 4 Michaelis-Menten analysis

The enzyme kinetics of α-amylase action on starch are complex (Butterworth et al., 2011; Dona, Pages, Gilbert, & Kuchel, 2010; Walker & Whelan, 1960). In addition to the effects of granule and molecular structure of starch on catalysis (see above), the structure and physical properties of the food itself can affect the access of enzyme to the starch substrate. Encapsulation, for example, within intact plant food matrices hinders amylolysis (Edwards et al., 2014; Edwards, Warren et al., 2015; Edwards, Ryden et al., 2021). Encapsulation may also limit gelatinisation of starch during cooking/food processing by preventing water uptake and swelling of granules (Edwards et al., 2014; Edwards, Warren et al., 2015; Xiong et al., 2018) (Fig. 1).

Given the complexity of starch structure and the knowledge that α-amylase can attack a starch α-1-4 linked chain at numerous sites within the chain, there is no certainty that each site is equally reactive given any constraints introduced by an ordered starch structure. Therefore, the initial reaction velocities on which the Michaelis-Menten model is based cannot necessarily be extrapolated to the hydrolysis kinetics for the whole of the starch substrate. Hence the interest in extended hydrolysis investigations discussed in the section below dealing with digestibility curves.

Nevertheless, it has been known for many years that the relation between starch concentration [S] and the initial rates of digestion by amylase [v] can be fitted by the familiar Michaelis-Menten equation (Copeland, 2000):

(1) $$v=V_{max}\;S/\left(K_{m}+S\right)$$

**Supplemental Figure 1:**
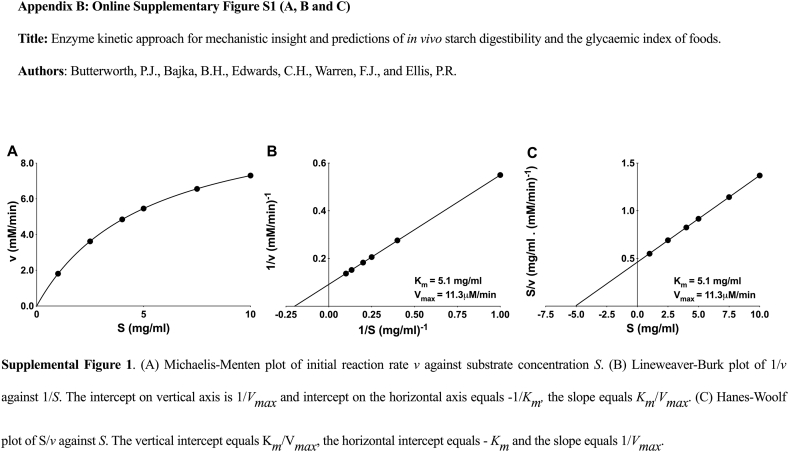
(A) Michaelis-Menten plot of initial reaction rate *v* against substrate concentration *S*. (B) Lineweaver-Burk plot of 1/*v* against 1/*S*. The intercept on vertical axis is 1/*V*_*max*_ and intercept on the horizontal axis equals -1/*K*_*m*_ the slope equals *K*_*m*_/*V*_*max*_. (C) Hanes-Woolf plot of S/*v* against *S*. The vertical intercept equals *K*_*m*_/*V*_*max*_, the horizontal intercept equals - *K*_*m*_ and the slope equals 1/*V*_*max*_.

The maximum rate*, V*_max_, is reached when the enzyme is saturated with its substrate (Copeland, 2000) (Appendix B: Supplemental Fig. S1A).

### 4.1 Fitting of kinetic data

The use of software for fitting to a rectangular hyperbola by non-linear regression allows determination of *V*_max_ and of the Michaelis constant, *K*_*m*_, which is the substrate concentration at which the catalytic rate proceeds at one half of the maximum, (i.e., *v* = *V*_max_/2). *V*_max_ is directly related to the total enzyme concentration [E] and can be written as *k*[E] where the proportionality constant *k* is frequently referred to as the catalytic rate constant (*k*_cat_) with dimensions of Time^−1^.

Because of substrate depletion during enzyme assays and competitive inhibition of amylase by its product maltose, an integrated form of the Michaelis-Menten equation incorporating a function for competitive inhibition has been recommended for data analysis (Dona et al., 2010). Maltose is a weak inhibitor (Seigner, Prodanov, & Marchis-Mouren, 1985; Warren, Butterworth, & Ellis, 2012) but some inhibition might occur during very lengthy digestion periods and/or if the starch is trapped within a food bolus allowing localised accumulation of product. If initial reaction rates are determined over relatively short time periods, however, the effects of substrate depletion and inhibition by accumulated maltose can be ignored.

A popular method of data analysis involves a fit to the double reciprocal (Lineweaver-Burk) form of the Michaelis-Menten equation:

(2) $$1/v=1/V_{max}+K_{m}/V_{max}\;\left[S\right]$$

although an alternative form of the equation commonly attributed to Hanes and Woolf is a preferred option:

(3) $$S/v=S/V_{max}+K_{m}/V_{max}$$

Lineweaver Burk plots (Appendix B: Supplemental Fig. S1B) place emphasis on data obtained at the lowest substrate concentrations with corresponding low reaction velocities, which are likely to be subject to the greatest experimental error (Copeland, 2000). Any error in *v* will be magnified when the reciprocal is taken. In Hanes-Woolf plots of *S/v* against *S* (Appendix B: Supplemental Fig. S1C) any errors are smaller, and the plot is therefore deemed preferable (Cornish-Bowden, 2004). Plots of 1/*v* against 1/*S* and/or *S*/*v* against S can be appropriate for published articles because of their familiarity to readers but should never be used for reliable estimation of the kinetic constants *K*_*m*_ and *V*_max_. Data should be fitted directly to the Michaelis-Menten equation by non-linear regression using freely available computer software.

### 4.2 Usefulness of kinetic parameters K_m_ and k_cat_/K_m_

The expression *k*_*cat*_*/K*_*m*_ is known as the specificity constant and/or the catalytic efficiency (CE). This second-order rate constant relates the reaction rate to the concentration of free enzyme. CE allows comparison of the relative rates of reaction of different substrates that are acted upon by the same enzyme. For amylase, starches of different botanical and/or food sources exemplify varieties of substrate for the enzyme. If the concentration of enzyme is unknown, *k*_*cat*_ cannot be determined directly, but *V*_max_*/K*_*m*_ values can be used as indicative of the respective CE of amylase for each substrate, provided that differences in the amount of enzyme (units of activity etc.) used for each substrate are accounted for. Because of variations in starch structure arising from botanical source and/or hydrothermal processing, CE values obtained *in vitro* of different starch forms can provide explanations for the known variations of *in vivo* digestion rates of different starchy foods. Michaelis-Menten studies of CE have yielded useful data on the relative ease of digestion of different botanical starches (Table 1) and of how *K*_*m*_ values decrease markedly after gelatinisation by up to 15-20-fold (Slaughter et al., 2001; Tahir et al., 2011). The fractional decrease in *K*_*m*_ as starch is subjected to hydrothermal treatments can monitor the degree of gelatinisation i.e., the loss of semi-crystallinity and conversion to a disordered structure. Also, changes in the relative *K*_*m*_ value may also be indicative of increased binding affinity of amylase to starch (Table 1) (Baldwin et al., 2015; Slaughter et al., 2001; Tahir, Ellis, & Butterworth, 2010; Tahir et al., 2011).

**Table 1:** Relationship of dissociation constant and *K*_*m*_ value for amylase binding to raw starches (data taken from Baldwin et al., 2015; Tahir et al., 2010; Warren et al., 2013; and Warren et al., 2011). CE values were determined at 37 °C but *K*_*d*_ at 0 °C. Values are means ± standard errors.

Starch type	*K*_*d*_ (mg/mL)	*K*_*m*_ (mg/mL)	*K*_*m*_*/K*_*d*_	CE (*k*_*cat*_*/K*_*m*_) x 10^−4^
Wheat	0.31 ± 0.03	8.4 ± 0.0	27.097	0.30 ± 0.0
Potato	1.26 ± 0.07	36.4 ± 8.3	28.89	0.08 ± 0.02
Waxy rice	0.41 ± 0.03	7.3 ± 1.5	17.39	0.52 ± 0.12
Pea (wild type)	0.81 ± 0.09	22.4 ± 1.1	27.65	0.17 ± 0.013
Pea *r* mutant	0.17 ± 0.02	2.8 ± 0.02	16.47	1.27 ± 0.13
Mean value (±standard error) of *K*_*m*_*/K*_*d*_ ratio of these starches			23.50 ± 2.70	

### 4.3 Binding studies

Direct binding studies and measurements of glycan chain flexibility by solution state NMR (Baldwin et al., 2015; Warren, Butterworth, & Ellis, 2013; Warren, Royall, Gaisford, Butterworth, & Ellis, 2011) have demonstrated the key importance to amylolysis of the initial binding interaction between amylase and starch. The binding seems to play a major role in the rate limiting step of the reaction in that studies performed on different botanical starches show that a direct relationship exists between the measured *K*_*m*_ values and the equivalent dissociation constants (*K*_*d*_) for starch-amylase complexes (Warren et al., 2011). For several different botanical sources of starches, the mean value for the *K*_*m*_/*K*_*d*_ ratio was 23.5 ± 2.7 (mean ± standard error; see Table 1). The similarity of the ratio values reveals that the binding step is a key factor in catalysis by α-amylase.

### 4.4 Inhibition studies

**Supplemental Figure 2:**
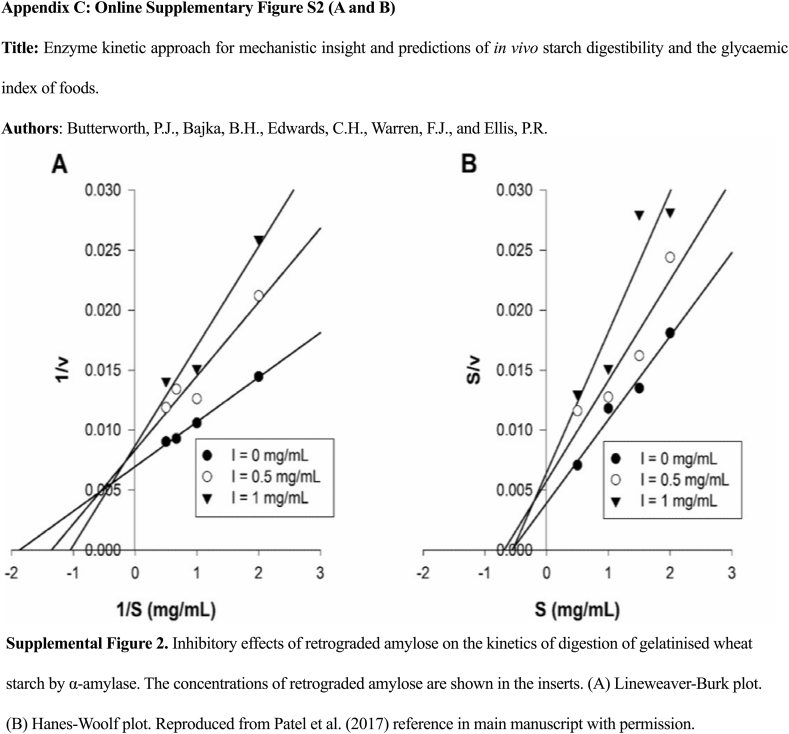
Inhibitory effects of retrograded amylose on the kinetics of digestion of gelatinised wheat starch by α-amylase. The concentrations of retrograded amylose are shown in the inserts. (A) Lineweaver-Burk plot. (B) Hanes-Woolf plot. Reproduced from Patel et al. (2017) reference in main manuscript with permission.

Extensions of the Michaelis-Menten equation to account for the effects of reversible inhibitors (Slaughter, Ellis, Jackson, & Butterworth, 2002) have been usefully applied in studies of α-amylase. This includes evidence obtained for the binding to the enzyme of the non-starch polysaccharides, guar galactomannan (Slaughter et al., 2002) and cellulose (Dhital, Warren, & Gidley, 2015), plus from studies of the effects of retrograded starch on amylase action (Patel et al., 2017) (Appendix C: Supplemental Fig. S2) and for inhibition of amylolysis by natural polyphenolic compounds (Lo Piparo et al., 2008; Sun, Warren, Netzel, & Gidley, 2016). Determination of inhibitor *K*_*i*_ values (the dissociation constant for enzyme-inhibitor complexes) can identify and estimate the potency of dietary materials that could slow intestinal digestion of starch and attenuate postprandial peaks in glycaemia. Some authors prefer to express inhibition data in terms of IC_50_ values (i.e., the concentration of inhibitor that results in 50% inhibition of amylase activity) to indicate the inhibitory efficacy of an agent at concentrations likely to be experienced *in vivo*. Use of IC_50_ values may be well intentioned but unless it has been established that the inhibitor does not act competitively (either fully or in part), IC_50_ values determined *in vitro* will be dependent on the substrate concentration used in the enzyme assays (Sun et al., 2016). Additionally, *in vitro*-derived IC_50_s become somewhat arbitrary due to difficulties in estimating concentrations of starch and inhibitor(s) in the intestinal lumen.

## 5 Digestibility curves

A starch digestibility curve, and perhaps the first to be published, appeared in a 1967 publication by Robyt and French (1967). This paper focused on the mechanism of action of α-amylase rather than its nutritional significance. The current situation is very different. Digestibility curves are extremely common in published nutritional science and following methods popularised by Englyst and colleagues (Englyst, Englyst, Hudson, Cole, & Cummings, 1999; Englyst et al., 2018), the percentages of the total starch content that are digested by 20 min and 120 min are used for calculation of rapidly digested starch (RDS) and slowly digestible starch (SDS), respectively, and any starch remaining undigested is classed as resistant (RS). The classification of RDS and SDS, based on the degree of digestion at particular time points, was devised to approximate the physiological changes in glycaemia observed *in vivo* following dietary starch loads. From estimations of RDS and SDS defined in this way, predictions of likely *in vivo* glycaemic indices (GI) of various carbohydrate-containing foods have been made (Bornet et al., 1989; Englyst et al., 1999; Englyst et al., 2018). GI values of foods provide a basis for dietary advice given the association of conditions such as type 2 diabetes, with long-term consumption of diets rich in high GI foods that can bring about exaggerated glycaemic and insulinaemic responses leading to insulin resistance (Augustin et al., 2015; Jenkins et al., 1981, 2002; Livesey et al., 2019). Despite its popularity and simplicity, the Englyst method (Englyst et al., 1999, 2018) for determining and classifying starch into RDS and SDS fractions suffers from a serious misinterpretation of the chemical kinetics of amylolysis and so there are firm grounds (enzymological and practical) for recommending the use of rigorous alternative methods (Dhital et al., 2017).

### 5.1 Pseudo first-order kinetics of reaction

An important publication by Goñi, Garcia-Alonso, and Saura-Calixto (1997) showed that timed digestibility curves of cooked starches could be described by pseudo first-order kinetics. The ‘pseudo’ term arises because the reaction between starch and amylase is actually bimolecular. If the concentration of one of the reactants, amylase in this case, is kept constant any change in reaction rate with time is dependent only on the concentration of starch and the reaction can be treated kinetically as first-order. The rate at any time point will be directly proportional to the concentration of digestible (i.e., available) starch, but this concentration decreases as it becomes converted to products. Therefore inevitably, the reaction rate decreases. Thus, unless the concentration of starch is extremely high so that the fall in concentration during amylolysis is a negligible fraction of the total, the rate measured at 20 min is always going to exceed the rate at 120 min irrespective of whether there are differences in the intrinsic reaction rates of different starch fractions. Hence, the use of more rigorous kinetic analysis will be more reliable than the estimates of RDS and SDS by the Englyst method. *In vivo*, considerable depletion of the starch concentration is likely because of the very high activity of α-amylase in the small intestine (Butterworth et al., 2011).

Improved methods simply require determination of a larger number of experimental points during starch digestion rather than measurements at just the two time points for the Englyst method. In our experience, up to 10 experimental points with about 5 or 6 of these taken within the first 30 min of the digestibility period, are usually adequate. If the slope of digestibility plots is determined by assuming that adjacent data points are linearly related (Butterworth, Warren, Grassby, Patel, & Ellis, 2012), they need to be sufficiently close in time to meet the assumption of linearity. The extra work and cost involved may account for a reluctance to adapt the Englyst method, but a switch to rigorous analyses is more robust from a scientific viewpoint and can deliver richer information about the digestive properties of the starch or food materials under investigation (see below). An inter-laboratory validation of the Englyst method for determining RDS and SDS has been published (Englyst et al., 2018), but the validation involved ‘two-point’ assays (i.e., at 20 min and 120 min) performed by each contributing laboratory, and no comparison was made with data obtained by first-order kinetic analysis.

### 5.2 Log of slope (LOS) method, analysis and interpretation

Equation (4) shows the first-order rate equation that can be applied to starch digestibility (Goñi et al. (1997)):

(4) $$C_{t}=C_{\infty }\left(1-e^{-kt}\right)$$

*C*_*t*_ is the concentration of the reactant at time *t*, *C*_*∞*_ is the corresponding concentration at the end point of the reaction and *k* is a pseudo first-order digestibility rate constant with dimensions of reciprocal time and is an intrinsic property of the enzyme. The equation is usually transformed into a logarithmic form for analysis of digestibility data:

(5) $$Ln\left[C_{\infty }-C_{t}/C_{\infty }\right]=-kt$$

A plot of *Ln* [*C*_*∞*_ *– C*_*t*_*/C*_*∞*_] against *t* is linear with a slope of –*k*. Estimations of *k* and *C*_*∞*_ and GI measurements obtained *in vivo* for the range of starch sources were used to develop an empirical equation for prediction of GI values from the *in vitro* digestion data alone (Goñi et al., 1997).

The use of Equation (5) calls for an accurate knowledge of *C*_*∞*_. Many investigators assume that the percentage of starch digested at the point when the slope of digestibility curves has become zero and the curves appear flat is a reliable estimate of the end point from which the concentration of starch remaining can be calculated. This point has sometimes been interpreted as the reaction equilibrium but under the conditions of a digestibility experiment, the hydrolysis is essentially irreversible so the reaction cannot attain equilibrium (Butterworth et al., 2011; Dhital et al., 2017). The flattening occurs because the available substrate has become exhausted.

To reach the flattening stage, the incubations need to be run for some time with the possibility of complications arising from loss of amylase activity by denaturation and/or inhibition of catalytic activity by the accumulation of maltose. A reliable procedure that does not call for the concentration at the end point is available and involves a differentiated form of Equation (5) (Butterworth et al., 2012; Edwards et al., 2014; Poulson, Ruiter, Visser, & Iverson, 2003).

(6) $$dC/dt=C_{\infty }ke^{-kt}$$

The logarithmic form of Equation (6) is:

(7) $$Ln\left(dC/dt\right)=Ln\left(C_{\infty }k\right)-kt$$

**Fig. 3:**
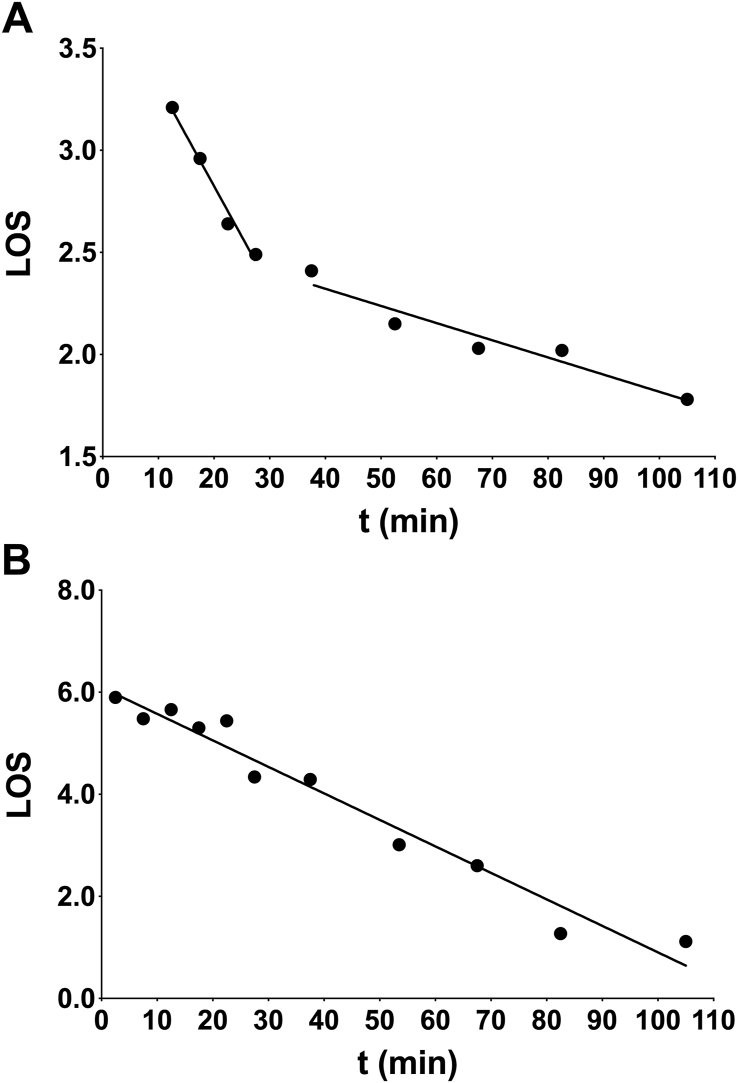
Log of slope (LOS) plots of wheat starch digestibility obtained with α-amylase at 37 °C. (A) Native (raw) starch and hydrothermally processed macroparticles of plant cells containing starch produce LOS plots with discontinuities. (B) Starches that are bioaccessible and gelatinised by hydrothermal treatment at 100 °C produce single LOS plots. The slope equals *–k* (digestibility constant) and the intercept on the vertical axis equals *Ln(C*_*∞*_*.k)*. Details of the LOS plot analysis and interpretation of the plots are published elsewhere (Butterworth et al., 2012; Edwards et al., 2014).

The term (*dC/dt*) is the slope of the digestibility curve and so a plot of the estimated slopes against *t* at various time points is linear with a slope of *–k* and an intercept on the Y axis equal to *Ln(C*_*∞*_ *k)* (Fig. 3).

If data points are determined at relatively small intervals of time (see above), the plot between adjacent points can be regarded as linear for easy estimation of the slope at various time points throughout the digestibility curves (Butterworth et al., 2012; Edwards et al., 2014). This log of slope plot (LOS) enables determination of the end point *C*_*∞*_ without the need of extended incubations with their inherent risks of loss of catalytic activity. Prolonged exposure of most enzymes at raised temperatures is likely to lead to a loss of catalytic activity. If experimenters are using pancreatin preparations that contain proteases, there is an even greater chance of inactivation because of proteolysis of amylase. *C*_*∞*_ may also provide an approximate indication of the quantity of RS by subtraction from the total amount of starch added to the reaction mixture at the start of the incubation. In any experiment, the digestibility constant, *k*, will be directly proportional to the concentration (or units of activity) of α-amylase used in the reaction mixture. Therefore, when comparing the digestibility constants obtained for a variety of different starches and starch-rich foods, allowance must be made for any differences in enzyme concentration used in the series of experiments.

LOS plots are very sensitive to changes in the digestibility behaviour of the substrate so if a starch/food sample contains α-glucan fractions digested at different rates, discontinuities appear in linear plots and the relative amounts of the fractions and their susceptibility to amylolysis can be estimated (Butterworth et al., 2012; Edwards et al., 2014). Digestibility data obtained for native starch granules and hydrothermally-processed particles of starch-containing plant foods produces LOS plots with discontinuities (Fig. 3A). There is a relatively rapid early phase followed by a later slower phase, which some investigators may describe as representative of RDS and SDS, respectively. The differences in digestion rate can be attributed to differences in the availability of the starch within an intact granule or food matrix. Free, mobile starch chains at surfaces exposed to the bulk solution containing amylase will be readily digested and the number of such chains increases with gelatinisation (Baldwin et al., 2015), but α-glucan chains buried within the granule will not be digested until amylase diffuses into the granule through pores or imperfections in the granule structure. The diffusion rate is low and so the observed rate of starch digestion will therefore be accordingly slow (Dhital et al., 2017). A similar effect would be seen in a food matrix, such as legume or cereal tissue particles, where enzyme diffusion rates are also expected to be low (Edwards et al., 2014; Edwards, Grundy et al., 2015; Edwards, Ryden et al., 2021; Pallares Pallares et al., 2018). After gelatinisation of pure starch or starch in tissues, where cell walls are ruptured and the starch is bioaccessible, single LOS plots appear because the majority of polyglucan chains are exposed to the enzyme solution (Baldwin et al., 2015; Edwards et al., 2014) (Fig. 3B).

It has been suggested that the LOS plot method, based on estimates of starch hydrolysed at individual time points, is subject to experimental error and that differentiated equations can result in a loss of precision (Syahariza, Sar, Hasjim, Tizzotti, & Gilbert, 2013; Yu, Toa, & Gilbert, 2018). Also, when slope changes appear in LOS plots, estimates of the time point at which any change occurs can be subjective. Therefore, testing of the goodness to fit to digestibility curves by non-linear methods should be performed using *C*_*∞*_ and *k* values derived from the LOS plot (Edwards et al., 2014). Direct fitting to Equation (4) by non-linear least squares has also been stated as a preferred alternative for estimating *C*_*∞*_ and *k* values (Syahariza et al., 2013; Yu, Tao, & Gilbert, 2018). A comprehensive review of various methods that can be applied for modelling of digestibility curves has been published (Nguyen & Sopade, 2018), but most investigators are likely to find that the LOS method and/or direct fitting to a first-order equation are suitable for general use.

## 6 Predictions of postprandial glycaemia

Interesting as the enzyme kinetic studies are in relation to structure, properties and composition of starch and starchy foods, from a nutritional standpoint, how *in vitro* amylolysis studies may predict postprandial glycaemia and insulinaemia is of prime importance. Goñi and colleagues included GI determinations *in vivo* following ingestion of various starch-containing foods from which a relationship was derived for predicting GI values (Goñi et al., 1997). The area under the curve (AUC) of digestibility plots compared with that obtained for white bread (standardised as 100) was used to provide a Hydrolysis Index (HI) value. The authors claimed a linear correlation between the measured GI and HI values and generated an empirical equation linking the two parameters, but examination of the literature indicates that the principle does not seem to have been widely adopted.

AUC can be readily calculated by insertion of *k* and *C*_*∞*_ values derived from LOS plots into the integrated form of Equation (4) from time zero to time *t* (Equation (8)) (Butterworth et al., 2012):

(8) $$AUC=C_{\infty }t+\left(C_{\infty }/k\right)\left(e^{-kt}-1\right)$$

Evidence for the usefulness of HI determinations was obtained in a human ileostomy study conducted with test meals of wheat endosperm porridges (Edwards, Grundy et al., 2015). HI values calculated from AUC calculations, resulting from LOS fitting of *in vitro* digestibility curves obtained with finely milled endosperm flour to create a smooth porridge, were 33% higher than the equivalent values for coarse endosperm flour. The 33% difference correlated closely with differences in AUC of the glycaemic responses observed in the *in vivo* study of ileostomates after consumption of the smooth or coarse wheat endosperm (Edwards, Grundy et al., 2015). It was notable also that in the ileostomy volunteers, the blood glucose-dependent insulinotropic polypeptide concentrations, indicative of glucose absorption, differed in the test meals by an extent that was commensurate with the digestibility data obtained by LOS analysis.

More recently, it was reported that data obtained from digestibility curves and LOS analysis, correlated reasonably well with literature values of *in vivo* GI (Edwards, Cochetel, Setterfield, Perez-Moral, & Warren, 2019). However, indices for the extent of starch digested at 90 min (*C*_*90*_) and *C*_*∞*_ were the most strongly correlated with GI rankings for matched starchy food products. The authors concluded that the *in vitro* method for starch digestibility showed potential for rapid prediction of GI values.

## 7 Conclusions and final comments

A comparison of various kinetic methods, including the LOS model, for identifying fractions of starch of varying digestibility rate, has been published and the LOS system was reported to be generally reliable (Nguyen & Sopade, 2018; Yu et al., 2018). In spite of criticism of the Englyst RDS and SDS designations, their use is so widespread that it is unlikely that investigators will abandon these terms. Nutritionists continue to find use for the terms because proponents of the Englyst method claim that it provides reliable predictions of likely rises in glycaemia and insulinaemia after meal ingestion. Improved understanding, however, of starch structure and properties, and the kinetics of starch digestibility, should enable better estimates of RDS and SDS fractions in starch.

The relative proportion of RDS in starchy foods is often taken as indicative of how rapidly the starch will be digested *in vivo* and whether its consumption is likely to produce sharp peaks in glycaemia and insulinaemia. Recent developments in our understanding of amylolysis, however, emphasise the importance of basing estimations of RDS and SDS on sound data obtained from full digestibility curves rather than two time points of 20 and 120 min. A recent publication by Kim, Hong, Choi, and Moon (2021) supports the use of the RDS and SDS terminology and, like Sopade (2021, 2022), presents a modified form of LOS that considers simultaneous occurrence of rapid and slow digestion reactions with the rapid reaction predominating at the early stages. Compared with the original LOS method that assumes consecutive reactions (Edwards et al., 2014), Yu and colleagues also report on the likely occurrence of rapid and slow reactions proceeding in parallel (Yu, Zhou, & Li, 2021). The model of Kim et al. (2021) provided an improved fit to digestibility data obtained for a number of raw starches with better estimates of RDS and SDS. Previous findings however show that for raw starch granules, exposed and flexible polyglucan chains, the numbers of which are increased by hydrothermal processing, are targeted first by amylase (Baldwin et al., 2015). Bello-Perez, Agama-Acevedo, Garcia-Valle, and Alvarez-Ramirez (2019), suggesting that values for the slopes of digestibility curves at individual time points required for LOS plots, are best obtained by direct non-linear regression fitting of the digestibility data rather than by the method given by Butterworth et al. (2011).

*In vitro* digestibility measurements continue to be of considerable use in predicting *in vivo* glycaemia to allow identification and recommendation of suitable foods and diets for management of obesity and diabetes and for the development of novel ingredients and food products with enhanced nutritional qualities. The importance and potential significance of amylase measurements has been emphasised in a recent publication that relates the value of *in vitro* screening of digestibility profiles of food products for prediction of likely glycaemic responses *in vivo* (Edwards et al., 2019). It is becoming clear that a number of investigators have begun to adopt the LOS approach and introduce extensions that consider digestions that proceed at more than 1 or 2 distinct phases and hence allow improved predictions of likely GI outcomes (Sopade, 2021, 2022).

It is very important therefore, that methods used in the analysis of digestibility are rigorous. We hope that use of the kinetic methods described here means that digestibility data can be interpreted with greater precision and mechanistic insight, forming the basis of reliable nutritional opinion and future national dietary guidelines.

## CRediT authorship contribution statement

Peter J. Butterworth: Conceptualisation and development of review, and writing original draft and subsequent revisions; Balazs H. Bajka: Contributed to review development, revisions of article and preparation of Figures; Cathrina H. Edwards: Contributed to review development, revisions of article and preparation of Figures, including art work for Fig. 1; Frederick J. Warren: Contributed to review development and revisions of article; Peter R. Ellis: Conceptualisation and development of review and contributed to revisions of article. All authors read and approved the final manuscript.

## Declaration of competing interest

The authors declare that they have no known competing financial interests or personal relationships that could have appeared to influence the work reported in this paper.
