# Supplementary material for: Enzyme kinetic approach for mechanistic insight and predictions of in vivo starch digestibility and the glycaemic index of foods
Source: Trends Food Sci Technol. 2022 Feb;120:254–64. doi: 10.1016/j.tifs.2021.11.015 (PMC8850932; doi:10.1016/j.tifs.2021.11.015)
Supplement: Multimedia component 1 [file mmc1.docx]

**Appendix A: Online Supplementary Material (α-Amylase Methods)**

**Title of Manuscript:** Enzyme kinetic approach for mechanistic insight and predictions of *in vivo* starch digestibility and the glycaemic index of foods.

**Authors**: Butterworth, P.J., Bajka, B.H., Edwards, C.H., Warren, F.J., and Ellis, P.R.

***In Vitro* Methods for** **α-Amylase Assays**

Pure preparations of α-amylase from human saliva and porcine pancreas are available from commercial suppliers although the porcine pancreatic enzyme is usually the least expensive option. Cruder preparations to simulate human pancreatic secretions are also available, such as porcine pancreatin that is preferred by some investigators because it contains lipases, proteases and amylase and therefore, it is argued, may better approximate intestinal digestion. Protein and lipid can be bound by starch and it is known that such binding can slow the rate of digestion by amylase (Dhital, Warren, Butterworth, Ellis, & Gidley, 2017). Therefore, pre-treatment of starch substrate to remove bound protein and lipid may mean that subsequent *in vitro* measurements of amylolysis may reflect the intestinal digestion of starch. Interpretation of experimental data obtained with impure enzyme preparations can be challenging because of difficulties in calculation of enzyme concentrations and complexity of the enzyme system resulting from a mixture of hydrolases in pancreatin. Thus, the use of pure amylase preparations for *in vitro* experiments can be recommended on a number of grounds with the addition, if desired, of a pre-treatment stage using protease and lipase. The α-amylase concentration in the lumen of the human small intestine is estimated to range from 5-15 nM, which would give rise to high levels of catalytic activity (Butterworth, Warren, & Ellis, 2015). A general discussion of methods for studying food digestion has been presented (Brodkorb et al., 2019; Minekus et al., 2014).

The most widely used substrate for α-amylase is starch itself, but some artificial substrates are commercially available that are useful in enzyme kinetic work for direct monitoring of the enzyme reaction. *p*-Nitrophenyl glycosides are suitable, (McCrosky, Chang, David, & Winn, 1982) but they tend to be expensive and have poor affinity (i.e., high *K_m_* values) for α-amylase unless the polyglucan chain contains at least 5 glucose residues. The active site of mammalian α-amylases accommodates five glucose residues and the accumulated free energy of binding resulting from occupancy of all the sites is important for effective catalysis (Brayer, et al., 2000; Seigner, Proganov, & Marchis-Mauren, 1987). Any *p*-nitrophenyl-glucose residue released by amylase action is hydrolysed by α-glucosidase added to the reaction mixture and the liberated *p*-nitrophenol can be continuously followed spectrophotometrically at ~400 nm. The method is suitable for monitoring amylase activity but is still somewhat indirect in that it employs added α-glucosidase to complete the release of nitrophenol from the reaction products.

If an experimenter wishes to monitor amylase activity directly, however, a commercially available assay kit can be recommended. Enzchek^(R)^ produce modified maize starch that is extensively labelled with a fluorophore and available from ThermoFisher Scientific (Waltham, MA 02451, USA). The fluorescence of the substrate is internally quenched because of the high degree of labelling but once fragments are released during amylolysis, strong fluorescent signals are produced which are readily detectable in a plate reader. Fluorescence assays are extremely sensitive (often at least x100 more sensitive than spectrophotometry) and relatively easy to perform and adaptable for microplate methods, to allow high throughput and cost-effectiveness.

The commonest assay methods rely on measurement of reducing power emanating from the rate of release of maltose or after conversion of the disaccharide to glucose by addition of maltoglucosidase. A very popular colorimetric method for measuring reducing sugar and which is very easy to perform, employs dinitrosalicylate reagent (DNS). The method lacks sensitivity however and is not ideally suited for monitoring the earliest stages of a digestion reaction where the concentration of generated reducing equivalents will be very low. The early stages of a digestion curve i.e., up to 20 minutes, can contain much useful information about the digestion process but is frequently overlooked by investigators because of insensitive detection methods such as the use of DNS. Measurements of initial rates of reaction are essential if the investigation involves analysis of reaction rates by application of Michaelis-Menten kinetics. Quantification of reducing sugar by Prussian blue methods (Moretti & Torson, 2008; Slaughter, Ellis & Butterworth, 2001) or by reaction with *p*-hydroxybenzoic acid hydrazide (*p*HBAH) can be very sensitive and readily adaptable for rapid throughput using 96 well microplates. Measurement of glucose by glucose oxidase following conversion of amylolysis reaction products to glucose by maltoglucosidase is a sensitive method. However, it is recommended that glucosidase is added after cessation of the amylase step because when the two enzymes are both present in a reaction mixture, activation of amylase can occur, resulting in a possibility of over-estimation of the initial rates of amylolysis (Warren, Zhang, Waltzer, Gidley, & Dhital, 2005).

**References**

Brayer, G.D., Sidhu, G., Maurus, R., Rydberg, E.H., Braun, C., Wang, Y., et al. (2000). Subsite mapping of the human pancreatic α-amylase active site through structural, kinetic and mutagenesis techniques. *Biochemistry,* *39*, 4778-4791. https://doi.org/10.1021/bi9921182

Brodkorb, A., Egger, L., Alminger, M., Alvito, P., Assunção, R., Balance, S., et al. (2019). INFOGEST static in vitro simulation of gastrointestinal food digestion. *Nature Protocol*, *14*, 991-1014. https://doi.org/10.1038/s41596-018-0119-1

Butterworth, P.J., Warren, F.J., & Ellis, P.R. (2011). Human α-amylase and starch digestion: An interesting marriage. *Starch/Stärke*, *63*, 395-405. https://doi.org/10.1002/star.201000150

Dhital, S, Warren, F.J., Butterworth, P.J., Ellis, P.R., & Gidley, M.J. (2017). Mechanisms of starch digestion by α-amylase – structural basis for kinetic properties. *Critical Reviews in Food Science and Nutrition*, *57*, 875-892. https://doi.org/10.1080/10408398.2014.922043

McCrosky, R., Chang, T., David, H., & Winn, E. (1982). *p*-Nitrophenylglycosides as substrates for measurement of amylase in serum and urine. *Clinical Chemistry*, *28*, 1787-1791. https://doi.org/10.1093/clinchem/28.8.1787

Minekus, M., Alminger, M., Alvito, P., Balance, S., Bohn, T., Bourlieu, C., et al. (2014). A standardised static *in vitro* digestion method suitable for food – an international consensus. *Food and Function*, *5*, 1113-1124. https://doi.org/10.1039/c3fo60702j

Moretti, R., & Torson, J.S. (2008). A comparison of sugar indicators enables a universal high-throughput sugar-1-phosphate nucleotidyltransferase assay. *Analytical Biochemistry,* *377*, 251-258. https://doi.org/10.1016/j.ab.2008.03.018

Seigner, C., Prodanov, E., & Marchis-Mauren, G. (1987). The determination of substrate binding energies of porcine pancreatic α-amylase by comparing hydrolytic activity toward substrates. *Biochimica et Biophysica Acta (BBA) – Protein Structure and Molecular Enzymology*, *913*, 200-209. https://doi.org/10.1016/0167-4838(87)90331-1

Slaughter, S.L., Ellis, P.R., & Butterworth, P.J. (2001). An investigation of the action of porcine pancreatic α-amylase on native and gelatinised starches. *Biochimica et Biophysica Acta (BBA) – General Subjects*, *1525*, 29-36. https://doi.org/10.1016/S0304-4165(00)00162-8

Warren, F.J., Zhang, B., Waltzer, G., Gidley, M.J., & Dhital, S. (2015). The interplay of α-amylase and amyloglucosidase activities in the digestion of starch in *in vitro* enzymic systems. *Carbohydrate Polymers*, *117*, 192-200. https://doi.org/10.1016/j.carbpol.2014.09.043
