# Supplementary material for: Enzyme kinetic approach for mechanistic insight and predictions of in vivo starch digestibility and the glycaemic index of foods
Source: Trends Food Sci Technol. 2022 Feb;120:254–64. doi: 10.1016/j.tifs.2021.11.015 (PMC8850932; doi:10.1016/j.tifs.2021.11.015)
Supplement: Multimedia component 2 [file mmc2.docx]

**Supplemental Figure 1**. (A) Michaelis-Menten plot of initial reaction rate *v* against substrate concentration *S*. (B) Lineweaver-Burk plot of 1/*v* against 1/*S*. The intercept on vertical axis is 1/*V_max_* and intercept on the horizontal axis equals -1/*K_m_* the slope equals *K_m_*/*V_max_*. (C) Hanes-Woolf plot of S/*v* against *S*. The vertical intercept equals *K_m_*/*V_max_*, the horizontal intercept equals - *K_m_* and the slope equals 1/*V_max_*.

**Supplemental Figure 2.** Inhibitory effects of retrograded amylose on the kinetics of digestion of gelatinised wheat starch by α-amylase. The concentrations of retrograded amylose are shown in the inserts. (A) Lineweaver-Burk plot. (B) Hanes-Woolf plot. Reproduced from Patel et al. (2017) reference in main manuscript with permission.
